# Structure of the virulence-associated protein VapD from the intracellular pathogen *Rhodococcus equi*

**DOI:** 10.1107/S1399004714012632

**Published:** 2014-07-25

**Authors:** Jean L. Whittingham, Elena V. Blagova, Ciaran E. Finn, Haixia Luo, Raúl Miranda-CasoLuengo, Johan P. Turkenburg, Andrew P. Leech, Paul H. Walton, Alexey G. Murzin, Wim G. Meijer, Anthony J. Wilkinson

**Affiliations:** a Structural Biology Laboratory, Department of Chemistry, University of York, Heslington, York YO10 5DD, England; b UCD School of Biomolecular and Biomedical Science and UCD Conway Institute, University College Dublin, Dublin, Ireland; c MRC Laboratory of Molecular Biology, Francis Crick Avenue, Cambridge Biomedical Campus, Cambridge CB2 0QH, England

**Keywords:** virulence factors, bacterial pathogenesis, *Rhodococcus equi*, VapD

## Abstract

*Rhodococcus equi* is a multi-host pathogen that infects a range of animals as well as immune-compromised humans. Equine and porcine isolates harbour a virulence plasmid encoding a homologous family of virulence-associated proteins associated with the capacity of *R. equi* to divert the normal processes of endosomal maturation, enabling bacterial survival and proliferation in alveolar macrophages. To provide a basis for probing the function of the Vap proteins in virulence, the crystal structure of VapD was determined. VapD is a monomer as determined by multi-angle laser light scattering. The structure reveals an elliptical, compact eight-stranded β-barrel with a novel strand topology and pseudo-twofold symmetry, suggesting evolution from an ancestral dimer. Surface-associated octyl-β-d-glucoside molecules may provide clues to function. Circular-dichroism spectroscopic analysis suggests that the β-barrel structure is preceded by a natively disordered region at the N-terminus. Sequence comparisons indicate that the core folds of the other plasmid-encoded virulence-associated proteins from *R. equi* strains are similar to that of VapD. It is further shown that sequences encoding putative *R. equi* Vap-like proteins occur in diverse bacterial species. Finally, the functional implications of the structure are discussed in the light of the unique structural features of VapD and its partial structural similarity to other β-barrel proteins.

## Abstract 2

VapD is one of a set of highly homologous virulence-associated proteins from the multi-host pathogen *Rhodococcus equi.* The crystal structure reveals an eight-stranded β-barrel with a novel fold and a glycine rich ‘bald’ surface.

## 1. Introduction   

*Rhodococcus equi* is a soil-borne bacterial pathogen that causes disease in a range of animals, including pigs, sheep and cattle. It is also an opportunistic pathogen among immune-compromised humans such as AIDS patients, in which it causes tuberculosis-like symptoms. However, it is most closely associated with disease in young horses, in which it causes a severe pyogranulomatous pneumonia (Muscatello *et al.*, 2007 ▶). Mortality in infected foals of up to six months of age can approach 80% if the disease is not treated, and even with antibiotic treatment mortality approaches 30%. There is currently no commercially available vaccine licensed to prevent disease caused by *R. equi* (Muscatello *et al.*, 2007 ▶).

It is believed that the major route of *R. equi* infection and disease transmission is through inhalation into the lungs of aerosolized dust formed from contaminated faeces as well as by aerosol transmission between foals (Muscatello *et al.*, 2009 ▶). In the lungs, the bacteria enter alveolar macrophages by complement-mediated phagocytosis (Hondalus & Mosser, 1994 ▶). The phagosome in which *R. equi* resides would normally undergo a process of maturation, acquiring degradative and microbicidal properties through sequential fusion with a series of endomembrane compartments (early endosomes, late endosomes and lysosomes; Scott *et al.*, 2003 ▶). However, *R. equi* is able to escape cell killing in the macrophage by preventing phagosome maturation to the phago­lysosome stage (Meijer & Prescott, 2004 ▶). Analysis of *R. equi*-containing phagosomes for the presence of protein markers associated with the different stages of the endocytic pathway showed that early endosome markers are acquired and lost normally, whereas the acquisition of some late endosome markers is delayed or abolished (von Bargen & Haas, 2009 ▶). The vATPase proton pump (required for phagosome acidification) is not acquired and there are alterations in the physical appearance of the phagosome (Fernandez-Mora *et al.*, 2005 ▶). Collectively, this points to a block between the early and late endocytic stages. Having diverted the cell’s destruction pathways, the *R. equi* cells begin to multiply within the membrane-enclosed vesicle and to exert cytotoxic effects. This leads to killing of the macrophage and dissemination of the pathogen through the body, notably to the gut.

The ability of *R. equi* to survive and replicate inside macrophages is linked to its possession of a large virulence plasmid (80 kbp). Strains cured of this plasmid are no longer virulent in foals or in mouse models of infection and cannot survive in macrophages cultured *in vitro* (Hondalus & Mosser, 1994 ▶; Giguère *et al.*, 1999 ▶). The plasmid contains a pathogenicity island with 26 coding sequences, including that for VapA (virulence-associated protein A). VapA was identified in early studies as a 15–17 kDa factor associated with virulence, against which antibodies in the serum of foals infected with *R. equi* invariably react (Takai, Sekizaki *et al.*, 1991 ▶; Takai, Koike *et al.*, 1991 ▶). It is the defining member of a family of proteins unique to the *R. equi* virulence-plasmid pathogenicity island, with the others being VapC, VapD, VapE, VapG and VapH (Takai *et al.*, 2000 ▶). All of these proteins have secretion signals and a number have been observed to be exported from the cell (Byrne *et al.*, 2001 ▶). VapA appears to be unique among the virulence-associated proteins of *R. equi* in being retained on the cell surface (Takai *et al.*, 1992 ▶; Tan *et al.*, 1995 ▶; Byrne *et al.*, 2001 ▶).

VapA is associated with all *R. equi* strains isolated from infected foals; moreover, deletion mutagenesis experiments have shown that the presence of the *vapA* gene is essential for intracellular growth of the bacterium in macrophages (Jain *et al.*, 2003 ▶). While this shows that VapA is required for virulence, this factor is not sufficient since VapA expression in the absence of the other virulence plasmid-encoded proteins does not confer virulence (Giguère *et al.*, 1999 ▶). A recent study found that VapA is required for diversion of the phagosome-maturation pathway and to prevent acidification of phagosomes (von Bargen *et al.*, 2009 ▶). *vapA*, as part of the *vapAICD* operon (Byrne *et al.*, 2008 ▶), is coordinately regulated with other genes of the pathogenicity island (Miranda-CasoLuengo *et al.*, 2011 ▶). Elevated temperature, low pH, oxidative stress and low levels of iron stimulate its transcription (Takai *et al.*, 1992 ▶, 1996 ▶; Benoit *et al.*, 2001 ▶, 2002 ▶; Jordan *et al.*, 2003 ▶).

Extensive studies over two decades have illuminated the genetics and microbiology of *R. equi* and defined the roles of the major virulence factor VapA and its homologues in disease. However, the mechanisms by which these proteins promote cell survival in the macrophage are not known. To provide further insight into the role of the Vap proteins in the virulence of *R. equi*, we sought to determine the three-dimensional structure of VapA by X-ray crystallography. Following unsuccessful attempts to crystallize VapA, we turned to other members of the Vap protein family. This led to crystals of VapD suitable for structure determination; here, we present the structure of a core fragment of VapD solved to 1.9 Å resolution. Sequence comparisons together with circular-dichroism data for VapA suggest that the other virulence proteins of *R. equi* have closely similar structures.

## 2. Methods   

### 2.1. Cloning, expression and purification of VapA and VapD   

Both VapA and VapD contain signal sequences. The N-terminal amino acid of mature VapA was experimentally determined as Thr32 (Tan *et al.*, 1995 ▶). Using the *SignalP* server (Petersen *et al.*, 2011 ▶), the N-terminal amino-acid residue of the mature VapD protein is predicted to be Gln31. For the sake of clarity, numbering of amino-acid residues throughout is based on mature proteins, not taking the signal sequences into account. Fragments of the *vapA* gene encoding residues 1–158 (VapA-full) and the *vapD* gene encoding residues 1–134 (VapD-full) were amplified by polymerase chain reaction (PCR) from genomic DNA of *R. equi* virulent strain 103S (de la Peña-Moctezuma & Prescott, 1995 ▶) using the primers listed in Table 1 ▶. The amplified fragments were purified, digested with restriction endonucleases *Nde*I and *Xho*I (New England Biolabs Inc.) and then inserted into the *Escherichia coli* expression vector pET-30a(+) digested with the same enzymes (EDM Millipore Chemicals; Table 1 ▶). For the production of the N-terminally truncated form of VapD (residues 20–134; VapD-core), PCR was performed using the primers listed in Table 1 ▶ and the pET-30a-VapD-full plasmid DNA as a template. The PCR product was inserted into the expression vector pET-22b by the In-Fusion method (Clontech Laboratories). These constructs directed the expression of full-length VapA (VapA-full), full-length VapD (Vap-full) and the N-terminally truncated form of VapD (Vap-core), each with the amino-acid residues LEHHHHHH attached at the C-terminus. Site-directed mutagenesis was carried out using the primers listed in Table 1 ▶ to substitute methionine residues at positions Leu100 and Val123 in VapD-core. The sequences of the constructs were verified by sequencing the plasmids using T7 promoter primer.

The expression strain *E. coli* BL21 (DE3) (EDM Millipore Chemicals) was used for the production of VapA-full, VapD-full and VapD-core. In each case, transformed cells were grown at 37°C in lysogeny broth supplemented with antibiotic to an optical density of 0.7–1.0, induced with 1 m*M* isopropyl β-d-1-thiogalactopyranoside and then cultured for a further 3 h at 37°C. Selenomethionine-substituted truncated protein (VapD-core-SeMet) was produced by the same method using expression strain *E. coli* B834 (DE3) with the cells cultured in minimal medium containing selenomethionine with 50 µg ml^−1^ ampicillin (Studier *et al.*, 1990 ▶). To purify VapD-full, VapD-core or VapD-core-SeMet, cells were harvested by centrifugation, resuspended in extraction buffer consisting of 20 m*M* HEPES pH 7.5, 500 m*M* NaCl, 20 m*M* imidazole, to which an EDTA-free protease-inhibitor cocktail tablet (Roche Diagnostics, USA) had been added, and lysed by sonication. Cleared lysate was applied onto a nickel-affinity chromatography column (GE Healthcare) which had been pre-equilibrated with binding buffer (20 m*M* HEPES pH 7.5, 500 m*M* NaCl, 20 m*M* imidazole), and protein was eluted with a linear imidazole gradient (20–500 m*M*). Eluted fractions were analysed by SDS–PAGE and those containing overexpressed protein (approximately 90% pure) were pooled and concentrated and then applied onto a 16/60 S75 Superdex gel-filtration column (GE Healthcare) which had been pre-equilibrated with 20 m*M* HEPES pH 7.5, 150 m*M* NaCl. Eluted fractions containing pure Vap protein (appearing as a single band on SDS–PAGE) were concentrated to approximately 20–40 mg ml^−1^ protein and stored at −80°C. VapA-full was purified by the same method using a buffer containing 50 m*M* Tris–HCl pH 8.5 instead of 20 m*M* HEPES pH 7.5.

### 2.2. Size-exclusion chromatography with multi-angle laser light scattering (SEC-MALLS)   

For determination of the oligomeric state of VapD-core and VapD-full, the proteins were analysed by SEC-MALLS. Samples of protein at concentrations of 2.5 and 4.0 mg ml^−1^ for VapD-core and VapD-full, respectively, were loaded onto a Superdex S75 10/300 gel-filtration column equilibrated at 0.5 ml min^−1^ with a mobile phase consisting of 50 m*M* Tris–HCl pH 8.0, 150 m*M* NaCl. The eluate was passed through an SPD20A UV–Vis detector, a Wyatt DAWN HELEOS II 18-angle light-scattering detector and a Wyatt Optilab rEX refractive-index monitor with the system driven by a Shimadzu HPLC system comprising an LC-20AD pump. The data were processed and molecular masses were calculated using the *Astra V* software (Wyatt) as described previously (Colledge *et al.*, 2011 ▶).

### 2.3. Circular-dichroism spectroscopy   

Circular-dichroism (CD) spectra were recorded at 20°C with a Jasco J-180 CD spectrophotometer using a 0.1 cm path-length quartz cell as described previously (Levdikov *et al.*, 2012 ▶). Experiments were carried out in 20 m*M* Tris–HCl buffer pH 7.5. The protein concentrations in the samples were 0.2 mg ml^−1^. Random error and noise were reduced for each spectrum by averaging three scans in the wavelength range 260–195 nm. The signal acquired for the buffer used for dilution of the proteins was subtracted from the spectra acquired for the proteins.

### 2.4. Limited proteolysis   

Limited proteolysis was carried out on VapD-full in 10 µl reaction mixtures with a final protein concentration of 1 mg ml^−1^ in 50 m*M* Tris–HCl pH 7.5–8.0, 50–150 m*M* NaCl. Chymotrypsin was added to give chymotrypsin:protein weight ratios of 1:50; 1:100; 1:200 and 1:400. The time of incubation was varied from 15 min to 2 h. Each reaction was quenched by adding 1–10 m*M* PMSF. The reaction products were analysed by SDS–PAGE.

### 2.5. Protein crystallization, data collection, structure solution and refinement   

Screening for crystallization conditions for VapA-full, VapD-full, VapD-core and VapD-core-SeMet was carried out using a robotic (Mosquito) nanolitre sitting-drop format with commercially available 96-well screens. Promising crystallization conditions, which were only obtained with the VapD protein constructs, were then optimized by hand using the hanging-drop vapour-diffusion method in a 24-well format. X-ray diffraction data were collected at 100 K at the Diamond Light Source experimental stations I02 and I04. Data sets were integrated using *XDS* (Kabsch, 2010 ▶) and were scaled/merged with *AIMLESS* (Winn *et al.*, 2011 ▶; Evans & Murshudov, 2013 ▶). The structure of VapD-core was solved by the single-wavelength anomalous dispersion method (SAD) with data collected at a wavelength optimized for the *f*′′ signal of selenium using heavy-atom phasing and density modification as implemented in *SHELXC*/*D*/*E* (Sheldrick, 2010 ▶). Automated model building was then carried out using *Buccaneer* (Cowtan, 2006 ▶). The resulting model, which constituted almost the entire protein chain, was refined against native data for VapD-core using maximum-likelihood methods as implemented in *REFMAC*5 (Murshudov *et al.*, 2011 ▶). This was interspersed with manual corrections to the model using *Coot* (Emsley *et al.*, 2010 ▶). The refined model was then used to solve and partially refine the structure of the (isomorphous) VapD-full to investigate the conformation of the N-terminus of the protein.

Multiple sequence alignment was performed using *ClustalW* (Thompson *et al.*, 1994 ▶), secondary-structure predictions were carried out using the *PSIPRED* protein structure-prediction server (McGuffin *et al.*, 2000 ▶) and three-dimensional structural alignments were made using secondary-structure matching as implemented in *Coot* (Krissinel & Henrick, 2004 ▶). Structural figures were generated using *CCP*4*mg* (McNicholas *et al.*, 2011 ▶).

## 3. Results   

### 3.1. Sequence comparisons and structure prediction   

**Figure 1:**
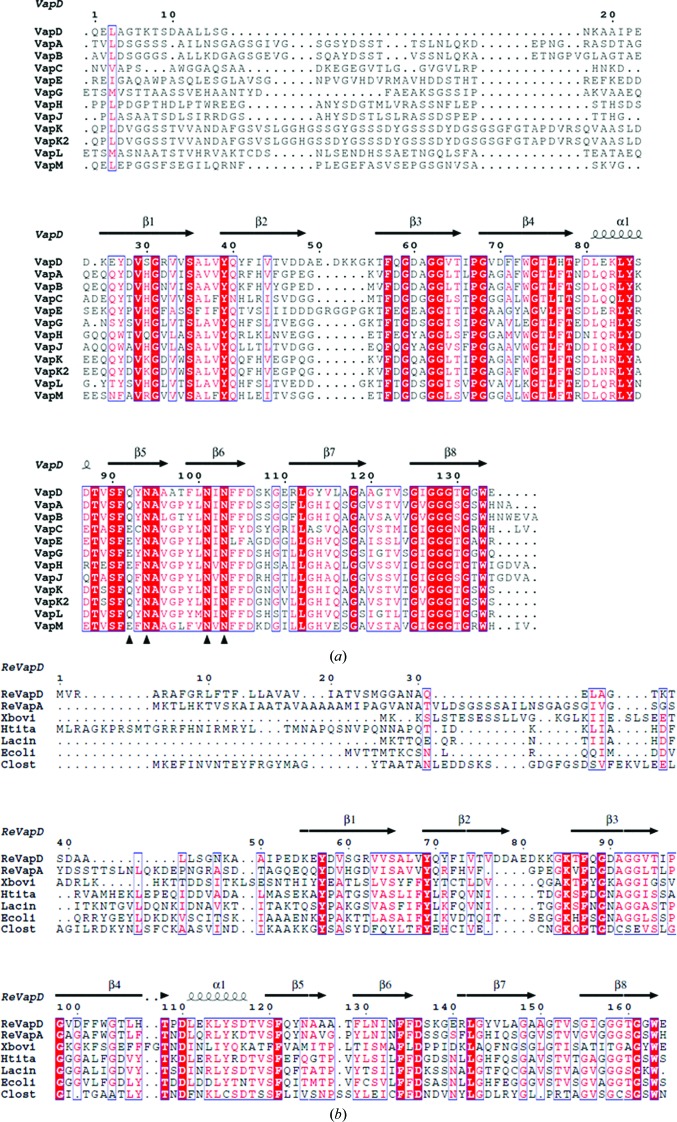
Comparison of Vap sequences. (*a*) Amino-acid residue sequence alignment of the mature Vap proteins encoded by the plasmids pVAPA1037 (Takai *et al.*, 2000 ▶) and pVAPB1593 (Letek *et al.*, 2008 ▶). Invariant residues in the alignment are shown in white on a red background; conserved residues are in blue boxes. The secondary structure for VapD is indicated above the alignment. Black triangles highlight four conserved amino-acid residues involved in the amide cluster referred to in the text. (*b*) Alignment of the sequences of *R. equi* Vap homologues from diverse species. The aligned sequences with their UniProt accession codes are VapD (ReVapD; B4F3C5) and VapA (ReVapA; B4F3C2) from *R. equi* and homologues from *Xeno­rhabdus bovienii* (Xbovi; D3V6D4), *Halomonas titaniciae* BH1 (Htita; L9UBC3), *Lacinutrix* sp. strain 5H374 (Lacin; F6GHM2), *Escherichia coli* H263 (Ecoli; 9VNI1) and *Clostridium* sp. DL-VIII (Clost; G7LYQ8). For this comparison the 30-residue signal peptide of VapD is retained, so the numbering is different to that used in (*a*).

An amino-acid sequence alignment of 12 virulence-associated proteins (Vaps) from two different host-specific virulence plasmids of *R. equi*, the horse-specific plasmid (pVAPA1037; VapA and VapC–I) and the pig-specific plasmid (pVAPB1593; VapB and VapJ–M), is shown in Fig. 1 ▶(*a*). These sequences share approximately 26% identity and 63% similarity with that of VapD, indicating that their structures are very similar. *In silico* secondary-structure analysis of the sequence of the Vap proteins predicted that the 20–50-residue variable regions following the signal peptides at their N-termini are natively disordered, while the remainder of the chains form β-strands and a single α-helix (McGuffin *et al.*, 2000 ▶). The sequence alignment highlights some interesting features including a conserved glycine-rich C-terminus and a 5–6-residue insert at residue 50 in VapD and VapE. This insert occurs between predicted secondary-structure elements and would not be expected to disrupt the overall fold. The Vap proteins have no homologues among proteins of known structure deposited in the Protein Data Bank and thus their three-dimensional structures cannot be predicted or modelled.

### 3.2. Expression, characterization and crystallization of VapD   

**Figure 2:**
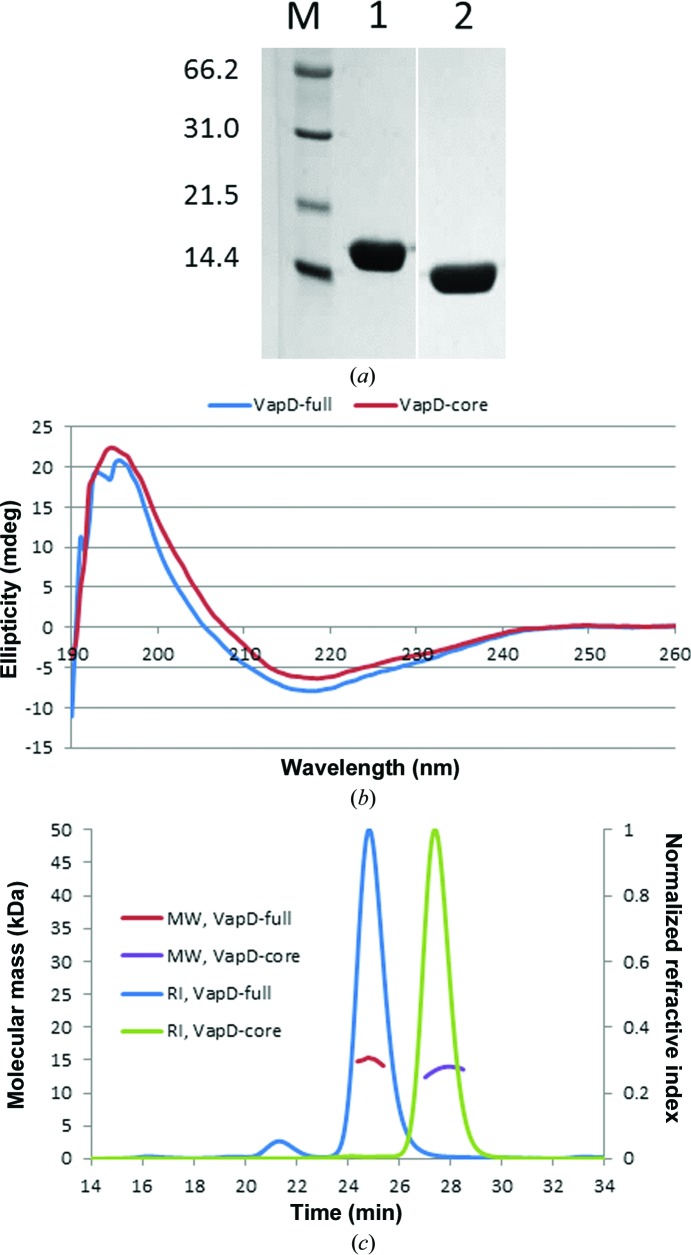
Physical properties of purified Vap proteins. (*a*) 15% SDS–PAGE analysis (molecular-weight markers 66.2, 31.0, 21.5 and 14.4 kDa) showing the effects of chymotrypsin digestion on VapD-full at a chymotrypsin:VapD weight ratio of 1:100. Lanes 1 and 2 are from the same gel, although intervening lanes have been cropped. (*b*) Circular-dichroism spectra of VapD-full and VapD-core. (*c*) SEC-MALLS analyses of VapD-full and VapD-core. The refractive index (RI) of the eluate from the Superdex S75 column is plotted as a function of time. For the principal peaks, the molecular weight (MW) of the species in the eluate is calculated from light-scattering measurements.

The *vapA* and *vapD* genes of *R. equi* encode 189-residue and 164-residue polypeptides, of which the first 31 and 30 residues, respectively, constitute a signal peptide that directs secretion of the proteins from the cell. For biochemical and structural characterization, the mature forms of VapA (residues 1–158; VapA-full) and VapD (residues 1–134; VapD-full) were produced in *E. coli* and purified by nickel-affinity chromatography followed by size-exclusion chromatography. The VapA protein preparations were often heterogeneous as judged by nondenaturing polyacrylamide gel electrophoresis and we were unable to crystallize this protein. VapD-full yielded weakly diffracting crystals. Although these crystals were initially unsuitable for structure determination, we focused our future efforts on VapD as it gave homogeneous protein preparations. In view of the predicted disorder at the N-terminus, a limited proteolysis experiment was carried out in the presence of chymotrypsin (Fig. 2 ▶ *a*). Electro-spray mass-spectrometric analyses of the protein before and after chymotrypsin treatment showed a molecular-mass difference of 1531 Da, indicating that cleavage had taken place after Leu14 to remove 15 amino-acid residues from the protein. Guided by this observation, a new construct was made to direct the production of VapD with a truncated N-terminus (VapD-core; Δ1–19).

The molecular characteristics of the full-length and core forms of VapD were assessed using circular-dichroism spectroscopy and size-exclusion chromatography with multi-angle laser light scattering (SEC-MALLS). In the CD spectrum, the molar ellipticity of VapD-full exhibits a shallow minimum at 215–220 nm consistent with the predominance of β-stranded structure (Fig. 2 ▶ *b*). The spectrum for VapD-core is very similar, suggesting that the structure is not significantly perturbed by the N-terminal truncation (Fig. 2 ▶ *b*). In the SEC-MALLS experiments, samples were fractionated on a gel-filtration column and the absorbance at 280 nm and the refractive index of the eluate were monitored together with the multi-angle laser light scattering of the sample. This enables the weight-average molecular weight (*M*_w_) of species in the eluate to be calculated continuously. Using this analysis, both constructs of VapD were shown to behave as monomers in solution, with experimentally determined molecular masses of 15.3 and 13.4 kDa for the full-length and core proteins, respectively (Fig. 2 ▶ *c*).

Truncation of the N-terminus of VapD and modifications to the crystallization conditions led to improvements in crystal size and, more importantly, diffraction quality. The VapD-core construct was then adapted to introduce internal methionine codons for the purpose of structure solution. Methionine substitutions were made at Leu100 and Val123, guided by the presence of Met residues at the corresponding positions of VapL and VapC, respectively (Fig. 1 ▶ *a*). Crystals of VapD-core and VapD-core-SeMet were obtained under similar crystallization conditions (Table 2 ▶). In both cases, octyl-β-d-glucoside proved to be an essential crystallization component.

### 3.3. Structure determination and refinement   

**Figure 3:**
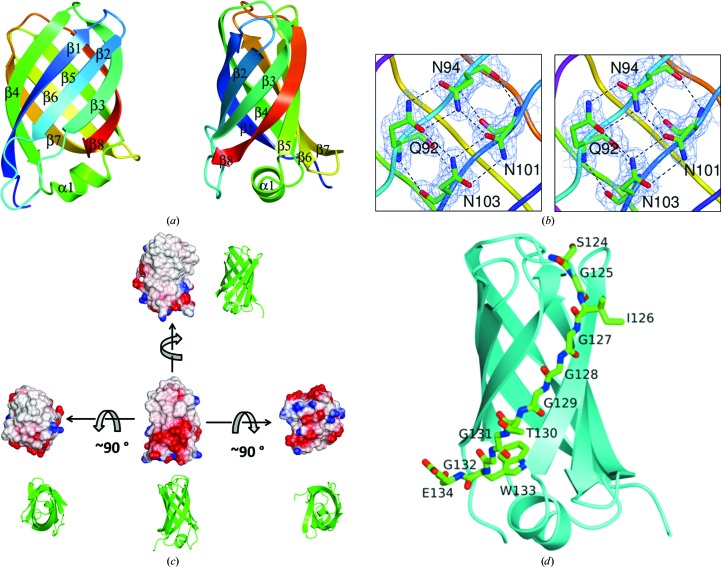
Structure of VapD. (*a*) Approximately orthogonal views of the VapD chain represented as a ribbon trace and colour-ramped from the N-terminus (blue) to the C-terminus (red). The secondary-structure elements are labelled. (*b*) Stereoview of the electron density (2*F*_o_ − *F*_c_) contoured at the 1σ level and displayed on the side-chain amide-containing residues, which form a closed hydrogen-bonding network on the surface of VapD. (*c*) Electrostatic surface rendering of VapD with positive electrostatic potential in blue and negative electrostatic potential in red. The extended apolar surfaces are evident. The orientation of the molecule in each case is apparent from the adjacent ribbon rendering. (*d*) The C-terminal residues of VapD: the chain is shown as a cyan ribbon with residues 124–134 in cylinder format coloured by atom and labelled. These and subsequent structural figures were produced using *CCP*4*mg* (McNicholas *et al.*, 2011 ▶).

Data collected from a single crystal of VapD-core-SeMet extending to 2.01 Å spacing (Table 2 ▶) were used to solve the structure by single-wavelength anomalous dispersion (SAD). Two Se sites were found using *SHELXD* (Sheldrick, 2010 ▶), consistent with the presence of one VapD molecule in the asymmetric unit of the crystal. The protein structure was then built and refined against native data extending to 1.9 Å spacing collected from a single crystal of VapD-core. During refinement, large peaks of positive difference electron density appeared indicating the presence of octyl-β-d-glucoside bound between the protein molecules. Three octyl-β-d-glucoside molecules were built into the structure, and a fourth potential site was identified but not modelled as the weakness of the electron density suggested low occupancy. The refined structure consists of residues Pro22–Glu134 (Fig. 3 ▶ *a*). Residues 20–21 at the N-terminus and the C-terminal His_6_ tag are not defined in the electron-density maps and are assumed to be disordered. The maps are otherwise of very good quality (Fig. 3 ▶ *b*). The modelled solvent content is lower than might be expected for a protein of this size owing to the bound octyl-β-d-glucoside molecules, which cover a significant proportion of the surface area of the protein.

At this point, following modifications to the crystallization and cryoprotection protocols, we were able to collect an X-ray diffraction data set extending to 2.1 Å spacing from a single crystal of VapD-full (Table 2 ▶). Preliminary refinement against these new data showed that residues preceding Pro22 were not defined in 2*F*_o_ − *F*_c_ and *F*_o_ − *F*_c_ electron-density maps and that the structural model could not be extended beyond that of VapD-core. These data are consistent with the prediction that the N-terminal 20 residues of VapD are natively disordered.

### 3.4. The protein fold of VapD-core   

The protein fold of VapD-core consists of a compact eight-stranded β-barrel which is elliptical in cross-section (Figs. 3 ▶ *a* and 3 ▶ *c*). The strand ordering for the barrel is β1–β2–β3–β8–β5–β6–β7–β4. At one end of the barrel the turns between strands are very short, giving rise to a smooth, rounded surface with a distinctly apolar character (Fig. 3 ▶ *c*). By contrast, the other end of the barrel has some longer inter-strand regions which protrude from the barrel in the form of a nine-residue α-helix with two flanking loops (β2–β3 and β6–β7; Figs. 3 ▶ *a* and 3 ▶ *c*). This more complex end of the protein is richer in charged and polar surface residues (Fig. 3 ▶ *c*). Notably, in the β2–β3 loop there is a group of acidic side chains (Asp47-Asp-Ala-Asp-Glu) followed by three basic side chains (Lys52-Lys-Gly-Lys). This loop-forming segment of the polypeptide appears as an insertion in the multiple sequence alignment that is restricted to VapD and VapE (Fig. 1 ▶ *a*). It is the least well ordered part of the VapD-core protein, as is shown by the lack of electron density for some of the side chains. Other parts of the protein surface are strikingly hydrophobic (Fig. 3 ▶ *c*).

The C-terminal segment of the polypeptide (residues 124–134) contains six glycines (Figs. 1 ▶ *a* and 3 ▶ *d*). As the side chains of Ser124, Ile126 and Glu134 project outwards, the only contribution to the protein core from this segment of the polypeptide is made by the side chain of Trp133.

The closed β-barrel architecture of the VapD fold can be classified by the number of strands *n* = 8 and the Shear number *S* = 10 (Murzin *et al.*, 1994*a* ▶). Barrels of this class are common and are found in both globular and membrane proteins. Barrels within the same class display similar geometrical features but may have different topologies (Murzin *et al.*, 1994*b* ▶). The topology of the VapD barrel has not been observed before. The strand order of its antiparallel β-sheet is β1–β2–β3–β8–β5–β6–β7–β4, which differs from the meander topology β1–β2–β3–β4–β5–β6–β7–β8 typical of this class of β-barrel proteins by the transposition of strands β4 and β8. There are three inter-strand connections capping the ends of the barrel: two (between strands β3 and β4 and strands β7 and β8) are at one end and one, containing the short α-helix (between strands β4 and β5), is at the other end. The VapD barrel is flattened in appearance. The strands β4 and β8 are strongly coiled to create ‘corners’ between ‘flat sides’ (Murzin *et al.*, 1994*b* ▶). The coiling of these strands is facilitated by conserved glycine residues (68, 74, 125, 127, 129 and 131) that adopt extended conformations with ϕ, ψ values outside the normally allowed β-sheet region. These ‘Gly kinks’ sharply change the directions of the strands but allow regular hydrogen bonding to be maintained on both sides of the kinked strands (Murzin *et al.*, 1994*b* ▶).

### 3.5. The core of the barrel   

**Figure 4:**
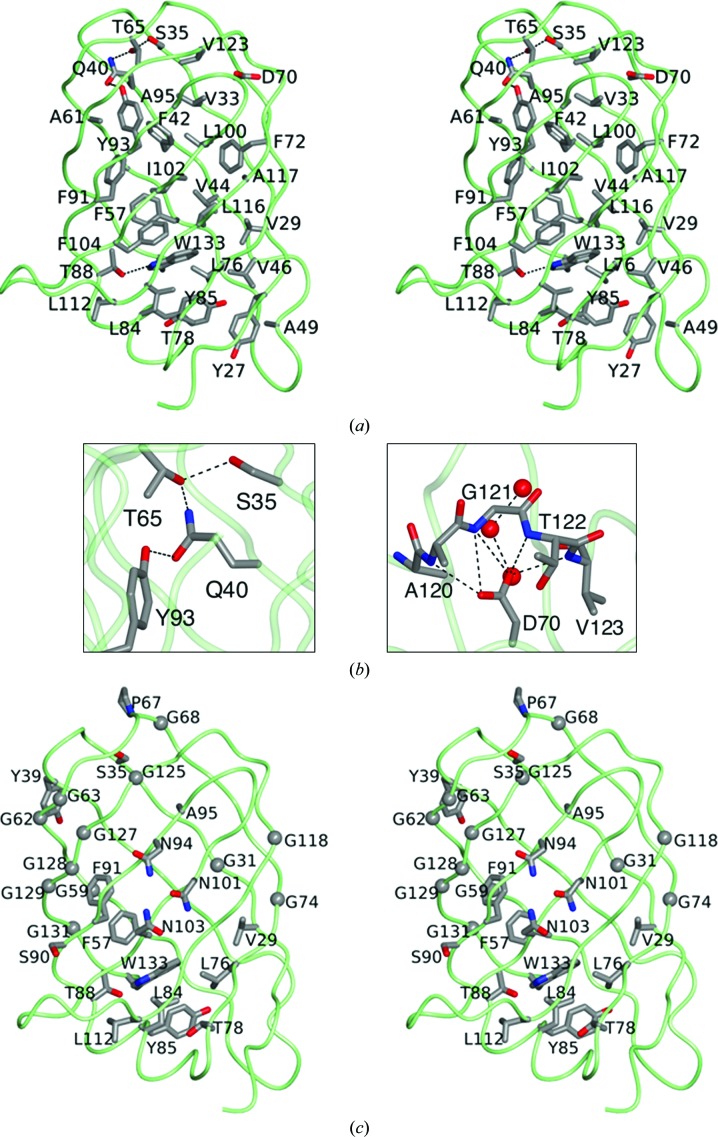
Core and conserved residues. (*a*) Stereoview of the VapD chain represented as a green worm highlighting the side chains of residues (which are labelled) in the protein core, defined here as residues with <10 Å^2^ of accessible surface area. The C^α^ atoms and side chains of these residues are shown in cylinder format coloured by atom: carbon, grey; oxygen, red; nitrogen, blue. Side chain–side chain hydrogen bonds are indicated by dashed lines. (*b*) Local hydrogen-bonding networks in the otherwise apolar core of VapD: left, interactions of the side chains of Ser35, Gln40, Thr65 and Tyr93; right, the role of Asp70 in organizing the β7–β8 loop through side-chain carboxylate–main-chain amide interactions with Ala120, Gly121, Thr122 and Val123. Also shown is the interaction of Asp70 with one of three buried water molecules. (*c*) View of the VapD chain as in (*a*) depicting the 30 invariant residues from the alignment of Vap proteins shown in Fig. 1 ▶. The C^α^ atoms of the invariant glycine residues are shown as spheres.

As a closed β-barrel structure, VapD has a dense core formed by the side chains of alternate residues on the β-strands, which project into the protein interior (Fig. 4 ▶ *a*). Trp133 is at the heart of this core, its bulky side chain forming apolar contacts to residues from six of the β-strands and the α-helix. In addition, its indole NH forms a hydrogen bond to the hydroxyl of Thr88. This is a rare polar interaction in a strikingly hydrophobic protein core. The side chain of Trp133 also packs between the rings of Tyr85 and Phe57, and this aromatic cluster extends to Phe104, Phe42, Phe91, Phe93 and Phe72.

The uppermost layer of the protein interior, when viewed as in Fig. 4 ▶(*a*), features a number of buried polar side chains, including those of Gln40, Ser35, Thr65 and Tyr93, that form a network of buried hydrogen bonds (Fig. 4 ▶ *b*). Although just five residues long, the β7–β8 loop is the longest of the loops at the upper end of the molecule. Asp70 appears to play an important role in satisfying the hydrogen-bonding requirements of the main-chain portion of this loop and in determining its conformation, as shown by the charge–dipole interactions between its side-chain carboxylate and the main-chain amide groups of the four successive residues Ala120, Gly121, Thr122 and Val123 (Fig. 4 ▶ *b*). The carboxylate also forms a polar interaction with the most buried member of a set of three water molecules that form a short channel to the surface. Despite its important structural role in VapD, this residue is not conserved in any of the other Vap proteins of *R. equi*.

### 3.6. Sequence considerations   

In Fig. 4 ▶(*c*), the invariant residues in the plasmid-encoded virulence-associated proteins of *R. equi* (Fig. 1 ▶ *a*) are mapped onto the structure of VapD. These residues are asymmetrically distributed in three dimensions, with the majority being located to the left of a diagonal running from the top left to the bottom right through the structure in the view shown in Fig. 4 ▶(*c*). There is a cluster of conserved residues with aromatic and aliphatic side chains that pack around Trp133 in the core, in addition to Thr88, whose side-chain hydroxyl forms a hydrogen bond with the indole NH.

Of the 30 invariant residues, 12 are glycines. Eight of these (three located on strand β3 and a further five located on strand β8) form something of a cluster and contribute to a rather featureless region of the surface. The hydrogen-bonded pair of glycine residues, Gly62 and Gly128, is of special note since nonglycine residues in these positions would have their side chains exposed. Such cross-strand pairs of glycine residues are very rare, presumably because they greatly decrease the stability of the β-sheet. The conservation of these two glycine residues in the *R. equi* Vap family sequences therefore suggests a functional role. The glycine pair contributes to a ‘bald’ spot on the side of the barrel which is devoid of side chains and is similar in size to the face of a porphyrin ring. The ‘bald’ spot in VapD has a smooth flat surface that constitutes a binding site for two octyl-β-d-glucoside molecules (see below), which were introduced in the crystallization solution. Interestingly, in the only other β-barrel structure containing such a cross-strand pair of ‘surface’ glycine residues that we have found so far, the histidine porin OpdC (PDB entry 3sy9), there are again two lipid molecules bound to the bald spot around Gly64 and Gly104 in chain *B* (Eren *et al.*, 2012 ▶).

The area around the ‘bald’ spot in VapD is made up of hydrophobic and uncharged polar residues. However, many of the polar groups here form hydrogen bonds to one another, effectively enhancing the nonpolar character of this extended area. In particular, most of the main-chain groups in the capping loops β3–β4 and β7–β8 and the β-turns β1–β2 and β5–β6 at one end of the barrel are hydrogen-bonded to each other or to conserved side-chain groups. Moreover, adjacent to the bald spot are the conserved residues Asn94, Asn101 and Asn103, which together with Gln92 in VapD form a closed network of hydrogen bonds (Fig. 3 ▶ *b*). Gln92 is conserved but not invariant, frequently being replaced by the iso­steric Glu residue in other Vap proteins (Fig. 1 ▶ *a*).

### 3.7. Crystal packing and octyl-β-d-glucoside binding   

**Figure 5:**
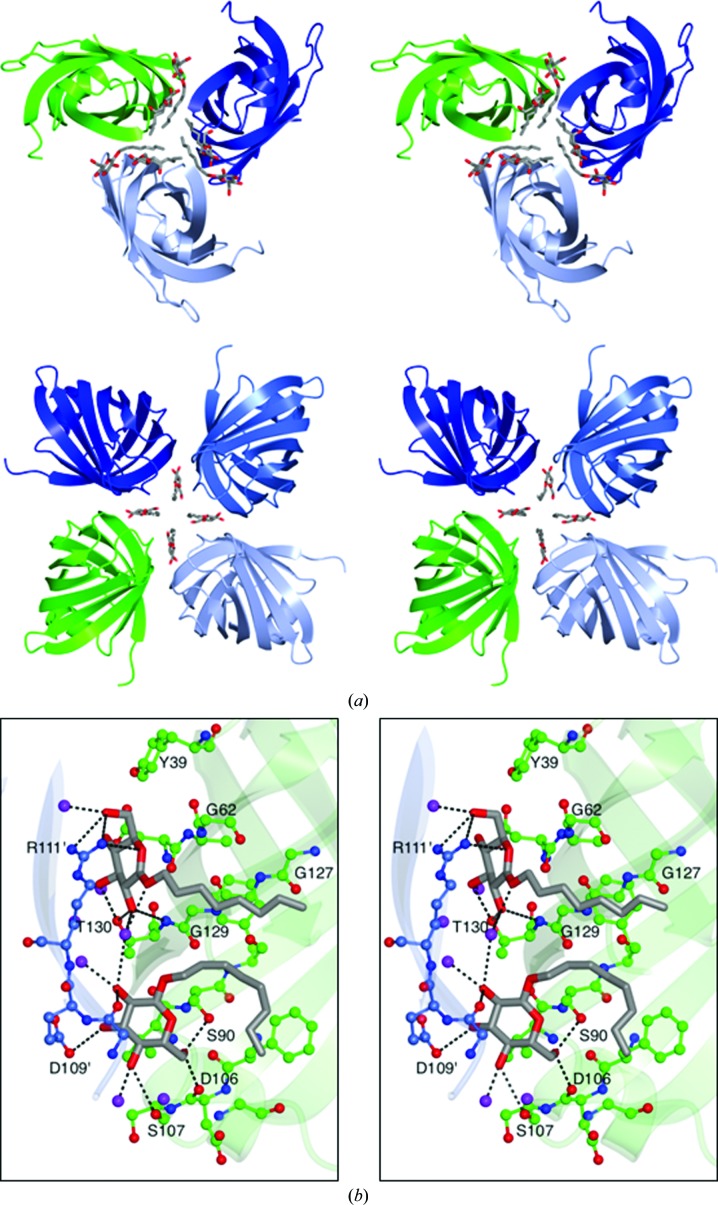
Octyl-β-d-glucoside binding and crystal packing. (*a*) Top, the packing of molecules around the crystallographic threefold axis with adjacent molecules in the lattice depicted as translucent ribbons coloured by chain. βOG1 and βOG2 are shown in cylinder format with C atoms in grey and O atoms in red. Bottom, packing of molecules around the crystallographic fourfold axis with βOG3 molecules represented as above. (*b*) Detail of the binding of βOG1 and βOG2. Residues surrounding the glycolipid are shown in ball-and-stick format coloured as above except that C atoms are shown in green for one VapD molecule and in light blue for its symmetry mate. Selected residues are labelled. Residues from the symmetry-related molecule are indicated by primes (′). Polar interactions are indicated by dashed lines.

VapD crystallizes in space group *F*432 with one molecule of protein per asymmetric unit and a solvent content of 43%. As mentioned above, the appearance of well diffracting crystals was dependent on the presence of octyl-β-d-glucoside (βOG) in the crystallization solution. As shown in Fig. 5 ▶(*a*), the three βOG molecules that were defined in the asymmetric unit of the structure mediate important contacts between molecules in the lattice. Two of these molecules, βOG1 and βOG2, make bridging interactions between VapD chains around the crystallographic threefold symmetry axes, generating clusters of six molecules (Fig. 5 ▶ *a*). Four βOG3 molecules assemble close to the crystallographic fourfold symmetry axes, where they similarly mediate lattice interactions (Fig. 5 ▶ *a*).

The interactions of βOG1 and βOG2 in the VapD crystal are shown in detail in Fig. 5 ▶(*b*). βOG1 resides between adjacent molecules, with the least-squares plane of its pyranose ring lying parallel to both the flat glycine-rich surface, featuring Gly62-Gly63 and Gly127-Gly128-Gly129, of one VapD chain and the planar guanidino group of Arg111 from a neighbouring molecule (Fig. 5 ▶ *b*). Multiple polar interactions are formed by the hydroxyl groups of the glucose and Thr130 from one VapD molecule and Arg111 of the neighbouring molecule. A water molecule makes bridging hydrogen bonds from the sugar of βOG1 to the 2-OH of βOG2, the 5-OH and 6-OH groups of which form hydrogen bonds to Ser107 and Ser90, respectively, while the 3-OH forms a charge–dipole interaction with the side-chain carboxylate of Asp109 in the neighbouring molecule. The eight-carbon aliphatic chain on βOG1 extends away from the sugar across the glycine-rich surface. The C_8_ chain of βOG2 initially packs beside that of βOG1 before folding back towards the sugar and packing across the surface of Phe104.

The sugar of βOG3 is somewhat disordered and does not form direct interactions with the protein molecules. Instead, the VapD interactions are mediated by the aliphatic chains which pack between the aromatic side chains of Phe71 and Trp73. The indole side chains of symmetry-related Trp73 side chains slot between the four βOG3s arranged around the fourfold axis.

### 3.8. The β-barrel structure of VapD is likely to be shared by VapA   

The sequence alignment shown in Fig. 1 ▶(*a*) suggests that the β-barrel structure of VapD will be shared by the other Vap proteins of *R. equi*, including the major virulence factor VapA. To provide experimental support for this assertion, we recorded a circular-dichroism spectrum of VapA (data not shown). Analysis of this spectrum using tools provided through the *DichroWeb* server (Whitmore & Wallace, 2008 ▶) suggests that VapA is rich in strands (45%) and turns (22%), with a small proportion of helix (7%). In addition, there is a significant proportion of unordered structure (26%). This spectrum is similar to that recorded for VapD and is consistent with the secondary-structure composition of the VapD crystal structure.

### 3.9. *R. equi* Vap-like proteins are widely distributed   

It was thought for many years that sequences encoding Vap proteins were restricted to *R. equi* strains harbouring virulence plasmids. However, when comparing the Vap sequences with recent entries in GenBank, it is apparent that *R. equi*-like *vap* genes are widely distributed. Thus, they occur in *E. coli*, *Clostridium* spp., *Halomonas* spp., *Lacinutrix* spp. and *Xenorhabdus bovienii*, representing the diverse phyla of Firmicutes, Proteobacteria and Bacteroidetes. The sizes of the encoded (putative) proteins fall within the range (150–206 residues) of those found in the *R. equi* Vaps, with sequence identities ranging from 27 to 42%. It is apparent from the alignment shown in Fig. 1 ▶(*b*) that, as for the Vap proteins of *R. equi*, the Vap homologues from these diverse species each possess a glycine-rich sequence followed by a tryptophan at their C-termini. However, none of these putative proteins has a recognisable secretion signal peptide at its N-terminus, suggesting that they may be cytoplasmic. The functions of the putative Vap protein homologues have yet to be determined. Interestingly, they are not plasmid-encoded; moreover, they appear to occur as single genes rather than as clusters of homologous genes as found in *R. equi*.

## 4. Discussion   

Infection and colonization of alveolar macrophages is a crucial step in the pathogenesis of *R. equi*-induced broncho­pneumonia in young horses. The precise mechanisms by which the pathogen evades the host cell’s defences are currently unknown, although studies suggest that *R. equi* is able to halt the normal cell-killing processes of the macrophage by preventing maturation of the phagosome at the early-to-late endocytic stage (Zink *et al.*, 1987 ▶; Fernandez-Mora *et al.*, 2005 ▶; Toyooka *et al.*, 2005 ▶).

The important but currently unknown modes of action of the Vap proteins in *R. equi* pathogenicity make them attractive targets for crystallographic studies. Given the high sequence similarity shared by these proteins, it is very probable that they exhibit the same essential structural features as VapD, including the eight-stranded β-barrel, the β4–β5 α-helix and the β6–β7 loop. VapE is the only other protein expected to have the protracted loop between strands β2 and β3 seen in VapD. In the case of VapE, this loop is glycine-rich (Fig. 1 ▶ *a*). The conserved C-terminal tryptophan residue is a dominant feature of this protein structure. This residue may have a functional role as well as being an essential structural component. For all of the Vap proteins, the hypervariable regions at the N-termini almost certainly lack ordered structure, although the variety in their length and sequence hints at different functionalities or different interacting partners.

### 4.1. Structure comparisons and functional implications   

**Figure 6:**
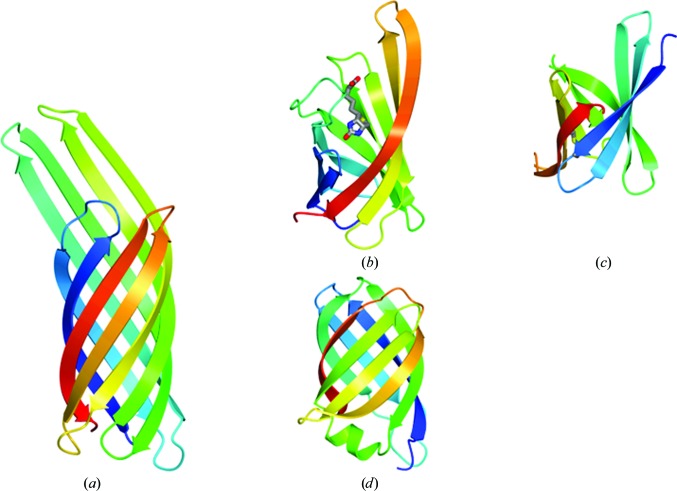
Selected β-barrel protein structures with partial similarity to VapD represented as ribbon traces and colour-ramped from the N-terminus (blue) to the C-terminus (red). (*a*) The archetypal eight-stranded β-barrel protein OmpX (*E. coli*). (*b*) Bradavidin (*B. japonicum*), *Q*-score = 0.20, for which 69 of 105 residues can be superposed onto VapD with an r.m.s.d. of 3.0 Å. (*c*) PliC (*S. typhimurium*), *Q*-score = 0.19, for which 62 of 84 residues can be superposed onto VapD with an r.m.s.d. of 3.2 Å. (*d*) VapD (*R. equi*). In (*b*) the biotin ligand is shown in cylinder format and is coloured by atom.

Despite its novel topology, the VapD fold shows extensive although partial similarity to other β-barrel folds that is detectable by popular structure-similarity search tools such as *DALI* and *PDBeFold*. The top-scoring hits are β-barrels of the same structural class (*n* = 8; *S* = 10) with simple meander topology, six of the eight stands of which (β2, β3, β4, β6, β7 and β8) can be structurally aligned with six strands in the VapD barrel (β1, β2, β3, β5, β6 and β7, respectively). Amongst these hits are β-barrel proteins implicated in bacterial virulence (Fig. 6 ▶).

One such protein, OmpX (Fig. 6 ▶ *a*; PDB code 1qj8; Vogt & Schulz, 1999 ▶), belongs to a family of membrane proteins that plays roles in (i) bacterial adhesion to, and entry into, mammalian cells and (ii) resistance to attack by the human complement system. Although the Vap proteins of *R. equi* are not membrane-spanning, the presence of bound βOG molecules in the crystal structure of VapD suggests the possibility of a transient association with the complex mycolic acid-rich cell envelope of this Gram-positive bacterium.

Bradavidin 2, an avidin-like protein from *Bradyrhizobium japonicum*, binds biotin in a cavity in the barrel interior with the β3–β4 loop serving as a lid (Fig. 6 ▶ *b*; Leppiniemi *et al.*, 2013 ▶). The absence of a cavity in VapD suggests that it is not involved in small-molecule binding. Curiously, in bradavidin and other avidin homologues ligand binding is associated with dimer and tetramer formation and in other instances biotin is bound at subunit interfaces. This is interesting in view of the mode of βOG1 and βOG2 binding in the subunit interfaces between pairs of VapD molecules in the crystal.

Like VapD, the periplasmic lysozyme inhibitor PliC from *Salmonella typhimurium* (PDB entry 3oe3; Leysen *et al.*, 2011 ▶; Fig. 6 ▶ *c*) is secreted through the cytoplasmic membrane, although in this Gram-negative bacterium PliC is retained in the periplasmic space. It is thought that PliC inhibits the bactericidal action of lysozymes which, following permeabilization of the outer membrane, degrade cell-wall peptido­glycan as part of the innate immune response of animals. The structural similarity, their extracellular localization and their functional association with bacterial virulence suggest the interesting possibility that the Vap proteins of *R. equi* may act as inhibitors of enzymes involved in endosome-associated host defence.

The unique structural features of VapD conserved in the Vap proteins of *R. equi* provide similar functional insights. The ‘bald’ spot and its extensive surrounding nonpolar area to which two βOG molecules are bound suggests the possibility that the *R. equi* Vap family members make functional interactions with large nonpolar surfaces. By analogy with the binding of antifreeze proteins to particular planes in ice crystals, the ‘bald’ spot may direct VapD and its relatives to ordered lipid structures. An example of such a structure is the monolayer formed by trehalose 6,6′-dimycolate, a glycolipid of mycobacteria and *R. equi* known to be a virulence factor (Hsu *et al.*, 2011 ▶; Schabbing *et al.*, 1994 ▶; Sydor *et al.*, 2013 ▶). Mycolic acids and their derivatives are the main components of the cell envelope of these bacteria. An attractive hypothesis is that the VapA family members facilitate reorganization of this envelope, for example by providing molecular surfaces suitable for the nucleation of the toxic TDM monolayer. Conversely, they may bind to ordered structures in the cell envelope and help to disrupt them.

Finally, the two surface residues Tyr39 and Ser90 form a contiguous patch with the ‘bald’ spot at Gly62 and Gly128, which is conserved not only in the *R. equi* Vap proteins but also in their relatives from other bacterial species. The role of the conserved hydroxyl groups of these tyrosine and serine residues is not clear, but they may contribute to the recognition of a larger ligand or receptor. Alternatively, they may be the sites of as yet unknown post-translational modifications.

### 4.2. Internal symmetry   

**Figure 7:**
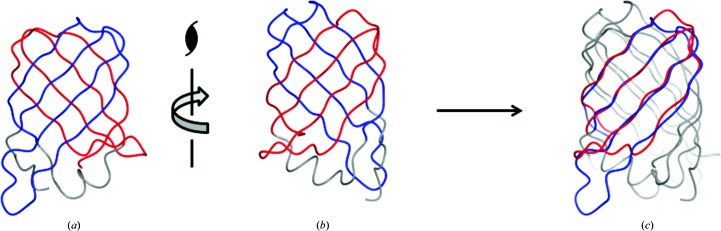
VapD and internal symmetry. VapD molecules are shown in worm representation with residues 29–77 coloured blue and residues 89–134 coloured red; the remaining residues are in grey. The C^α^ atoms of residues 29–77 of one molecule were superimposed onto residues 89–134 of a copy of this molecule using the *SSM Superpose* routine implemented in *CCP*4*mg*. (*a*, *b*) Views of VapD showing the orientation of the molecules before and after superposition and the direction of the screw axis that relates them. (*c*) The pair of superposed VapD molecules.

The proteins in the Protein Data Bank with the closest structural similarity to VapD have antiparallel β-barrel structures with the all-next-neighbour β1–β2–β3–β4–β5–β6–β7–β8 topology. This topology is clearly distinct from that of VapD, where the β1–β2–β3–β8-β5–β6–β7–β4 topology gives rise to multiple crossovers and a closed rather than an open barrel. As far as we are aware, this barrel topology has not been observed elsewhere and the VapD fold can thus be described as novel. Interestingly, the β-sheet topology apparent in VapD has pseudo-twofold symmetry. The two halves of the VapD barrel, from strand β1 to strand β4 (residues 29–77) and from strand β5 to strand β8 (residues 89–134), are structurally similar and can be superimposed onto each other with an r.m.s.d. of 1.8 Å for 42 pairs of C^α^ atoms (Fig. 7 ▶). This similarity extends to the turns and loops at one end of the barrel. This suggests the possibility that the VapD fold originated from an ancestral dimeric protein through the duplication of a gene encoding a four-stranded species followed by fusion.

In the VapD fold, the two halves of the molecule are related by a screw axis coinciding with the barrel axis. This is because twofold rotational symmetry about this axis is not compatible with Shear number *S* = 10 (Murzin *et al.*, 1994*a* ▶). This results in asymmetric interactions of the two capping loops (β3–β4 and β7–β8) at the end of the barrel. The β3–β4 loop effectively extends strand β3 by maintaining the regular hydrogen-bonding interactions with the beginning of strand β8. In contrast, the β7–β8 loop does not close the end of the barrel through main chain–main chain hydrogen bonding.

## 5. Conclusion   

In summary, the structure of VapD with its closed β-barrel does not immediately illuminate the function of the virulence proteins of *R. equi*, although unique aspects of its structure and its similarity to proteins of known function in other pathogens suggest future experiments. The binding of octyl-β-d-glucoside to the protein demonstrates the compatibility of the protein surface with glycolipids which are present in the outer membrane (Garton *et al.*, 2002 ▶). It is tempting to speculate that the hydrophobic portion of the barrel becomes buried in the unusual mycolic acid-rich outer surface of the Gram-positive *R. equi* bacterium. However, among the virulence-associated proteins of *R. equi*, only VapA remains associated with the cell surface; the others are secreted. Thus, it may be that the hydrophobic surface of VapD merely facilitates the passage of the protein through this complex layer.

## Supplementary Material

PDB reference: VapD, 4csb

## Figures and Tables

**Table 1:** Primers used in gene-cloning experiments

Protein construct/vector	Primers
VapA-full/pET-30a(+)	FWD 5-GGAATTCCATATGCCGTTCTTGATTCCCGG-3
	REV 5-CCGCTCGAGGGCGTTGTGCCAGCTACCAGA-3
VapD-full/pET-30a(+)	FWD 5-GGAATTC**CATATG**CAGGAGCTAGCTGGCACC-3
	REV 5-CCG**CTCGAG**CTCCCACCCGCCAGTGCC-3
VapD-core/pET-22b	FWD 5AAGGAGATATACATATGGCCATTCCTGAAGATAAAGAG-3
	REV 5-GGTGGTGGTGCTCGAGCTCCCACCCGCCAGTG-3
VapD-core(L100M/V123M)-SeMet/pET-22b	FWD_L100M 5-GCGGCCGCAACATTCATGAATATCAACTTTTTC-3
	REV_L100M 5-GAAAAAGTTGATATTCATGAATGTTGCGGCCGC-3
	FWD_V123M 5-GGTGCTGCTGGGACAATGTCGGGGATCGGTGGT-3
	REV_V123M 5-ACCACCGATCCCCGACATTGTCCCAGCAGCACC-3

**Table 2:** Crystallization conditions, data-collection and refinement statistics

	Se derivative	VapD-core native	VapD-full native
Crystallization conditions			
Protein solution†	1220mgml^1^ VapD-core-SeMet in 20m*M* HEPES pH 7.5, 500m*M* NaCl, 5m*M* TCEPHCl	20mgml^1^ VapD-core in 20m*M* HEPES pH 7.5, 500m*M* NaCl	12mgml^1^ VapD-full in 20m*M* HEPES pH 7.5, 150m*M* NaCl
Reservoir solution†	1.82.0*M* (NH_4_)_2_SO_4_, 0.2*M* NaSCN, 1.02.0%(*v*/*v*) MPD, 0.1%(*w*/*v*) OG	2.2*M* (NH_4_)_2_SO_4_, 0.2*M* NaSCN, 0.1%(*w*/*v*) OG	1.9*M* (NH_4_)_2_SO_4_, 0.1*M* bis-tris propane pH 5.66.0, 2.0%(*v*/*v*) MPD, 0.1%(*w*/*v*) OG
Data-collection statistics			
Diamond beamline	I04	I02	I04
Wavelength ()	0.97935	0.9795	0.97949
Space group	*F*432	*F*432	*F*432
Unit-cell parameter ()	*a* = 144.65	*a* = 142.84	*a* = 144.08
Resolution limits‡ ()	43.612.01 (2.062.01)	50.051.90 (1.941.90)	50.942.08 (2.142.08)
No. of unique reflections	8810	10376	8106
Completeness‡ (%)	96.5 (100)	99.7 (100)	99.3 (100)
Multiplicity‡	35.1 (39.0)	11.9 (12.0)	36.2 (37.3)
*R*_merge_ ‡ §	0.063 (0.598)	0.088 (0.833)	0.042 (0.571)
*I*/(*I*)‡	52.7 (8.4)	21.1 (3.7)	74.0 (8.9)
Refinement statistics			
*R*_cryst_ ¶/*R*_free_ ††		0.1608/0.1908	0.2182/0.2347 (refinement incomplete)
R.m.s.d., bond length (12)‡‡ ()		0.020 (0.020)	
R.m.s.d., angles‡‡ ()		2.141 (1.989)	
R.m.s.d., chiral volumes‡‡ (^3^)		0.224 (0.200)	
Average *B* (^2^)			
Protein		28.59	
OG		50.04	
Waters		48.57	
Ramachandran outliers (%)		0	

† Abbreviations: OG, octyl--D-glucoside; MPD, 2-methyl-2,4-pentanediol; TCEPHCl, tris(2-carboxyethyl)phosphine hydrochloride (reducing agent).

‡ Values in parentheses are for the highest resolution shell.

§ *R*_merge_ = 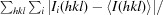 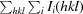, where *I*_*i*_(*hkl*) is the intensity of the *i*th measurement of a reflection with indexes *hkl* and *I*(*hkl*) is the statistically weighted average reflection intensity.

¶ *R*_cryst_ = 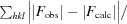 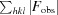, where *F*_obs_ and *F*_calc_ are the observed and calculated structure-factor amplitudes, respectively.

†† *R*_free_ is the *R*_cryst_ calculated with 5% of the reflections chosen at random and omitted from refinement.

‡‡ Average geometric restraints are given in parentheses.
